# Inhibition of vascular endothelial growth factor signaling facilitates liver repair from acute ethanol-induced injury in zebrafish

**DOI:** 10.1242/dmm.024950

**Published:** 2016-11-01

**Authors:** Changwen Zhang, Jillian L. Ellis, Chunyue Yin

**Affiliations:** 1 Division of Gastroenterology, Hepatology and Nutrition, Cincinnati Children's Hospital Medical Center, Cincinnati, OH 45229, USA; 2 Division of Developmental Biology, Cincinnati Children's Hospital Medical Center, Cincinnati, OH 45229, USA

**Keywords:** Hepatic stellate cells, Steatosis, Angiogenesis, Fibrogenesis, VEGF, Kdrl

## Abstract

Alcoholic liver disease (ALD) results from alcohol overconsumption and is among the leading causes of liver-related morbidity and mortality worldwide. Elevated expression of vascular endothelial growth factor (VEGF) and its receptors has been observed in ALD, but how it contributes to ALD pathophysiology is unclear. Here, we investigated the impact of VEGF signaling inhibition on an established zebrafish model of acute alcoholic liver injury. Kdrl activity was blocked by chemical inhibitor treatment or by genetic mutation. Exposing 4-day-old zebrafish larvae to 2% ethanol for 24 h induced hepatic steatosis, angiogenesis and fibrogenesis. The liver started self-repair once ethanol was removed. Although inhibiting Kdrl did not block the initial activation of hepatic stellate cells during ethanol treatment, it suppressed their proliferation, extracellular matrix protein deposition and fibrogenic gene expression after ethanol exposure, thus enhancing the liver repair. It also ameliorated hepatic steatosis and attenuated hepatic angiogenesis that accelerated after the ethanol treatment. qPCR showed that hepatic stellate cells are the first liver cell type to increase the expression of VEGF ligand and receptor genes in response to ethanol exposure. Both hepatic stellate cells and endothelial cells, but not hepatic parenchymal cells, expressed *kdrl* upon ethanol exposure and were likely the direct targets of Kdrl inhibition. Ethanol-induced steatosis and fibrogenesis still occurred in *cloche* mutants that have hepatic stellate cells but lack hepatic endothelial cells, and Kdrl inhibition suppressed both phenotypes in the mutants. These results suggest that VEGF signaling mediates interactions between activated hepatic stellate cells and hepatocytes that lead to steatosis. Our study demonstrates the involvement of VEGF signaling in regulating sustained liver injuries after acute alcohol exposure. It also provides a proof of principle of using the zebrafish model to identify molecular targets for developing ALD therapies.

## Abstract (precis)

**Summary:** Inhibition of VEGF signaling enhances recovery from acute ethanol-induced injury in the livers of zebrafish larvae, by directly suppressing fibrogenesis and angiogenesis, and indirectly ameliorating hepatic steatosis.

## INTRODUCTION

Alcoholic liver disease (ALD) is caused by acute or chronic alcohol abuse and affects more than 10 million people in the United States (Cohen and Nagy, 2011). Hepatic injury in ALD progresses in a systematic order. Steatosis, which is characterized by fat accumulation in the hepatocytes, occurs in the early stage of heavy drinking. Under the influence of genetic factors, viral infection, obesity and continuing alcohol intake, hepatic steatosis can progress to alcoholic hepatitis and eventually cirrhosis. The advances in our knowledge of ethanol metabolism and liver injury responses prompt the development of therapeutic approaches targeting cell death, inflammation, fibrosis and oxidative stress, yet the outcomes are far from satisfying. The cessation of alcohol use remains the most effective prevention and therapy for ALD (Louvet and Mathurin, 2015).

Elevated expression of pro-angiogenic factor vascular endothelial growth factor (VEGF) and its receptors, mainly FLT1 (also known as VEGFR1) and KDR (also known as VEGFR2), occurs in many forms of chronic liver diseases to induce pathologic angiogenesis (reviewed by Coulon et al., 2011; Elpek, 2015; Iwakiri et al., 2014). Upon liver injury, the vitamin-A-storing hepatic stellate cells (HSCs) transition into activated myofibroblast-like cells to generate scar tissue (Friedman, 2008). Activated HSCs upregulate expression of VEGF and its receptors (VEGFRs) in the rodent model of CCl_4_-induced liver fibrosis (Ishikawa et al., 1999). *In vitro*, increased expression of VEGF ligand and receptors in human and rat HSCs stimulates their proliferation, migration and chemotaxis in a paracrine and/or autocrine manner (Ankoma-Sey et al., 1998; Novo et al., 2007; Wang et al., 2004; Yoshiji et al., 2003). Blocking VEGF signaling either by treatment with a pan-VEGFR kinase inhibitor or by a neutralizing monoclonal antibody against VEGFR2 has an anti-fibrotic effect both *in vitro* and *in vivo* (Liu et al., 2009; Yoshiji et al., 2003). It has been reported that individuals with ALD have elevated plasma level of VEGFA (Kasztelan-Szczerbinska et al., 2014). In rodents, chronic ethanol exposure increases the hepatic expression of VEGF and VEGFR2 (Das et al., 2012; Raskopf et al., 2014). However, the exact role of VEGF signaling in ALD pathogenesis and progression has not been well characterized.

Although studies in the rodent ALD models have provided substantial insights into our understanding of the disease, there are limitations (reviewed by Louvet and Mathurin, 2015). Oral feeding of alcohol diet only causes steatosis in rodents (Ki et al., 2010; Tsuchiya et al., 2012). Development of inflammation and fibrosis requires a second insult (Koteish et al., 2002; Leo and Lieber, 1983). Chronic intragastric infusion results in more advanced liver damage but it is invasive and technically challenging (Tsukamoto et al., 1985, 2008). The teleost zebrafish show liver injury when exposed to ethanol in their water (Howarth et al., 2011; Jang et al., 2012; Lin et al., 2015; Passeri et al., 2009; Tran et al., 2015; Yin et al., 2012). Studying chronic alcoholic liver injury is difficult in adult zebrafish as they do not feed properly upon ethanol exposure (Goessling and Sadler, 2015). However, the larvae have been proven to be particularly useful for studying acute alcoholic liver injury (Howarth et al., 2011, 2013; Passeri et al., 2009; Yin et al., 2012). The zebrafish liver is functional and produces key enzymes for ethanol metabolism by 4 days post-fertilization (Lassen et al., 2005; Passeri et al., 2009; Reimers et al., 2004). Ethanol can be directly added to the water and is immediately ingested and metabolized by the larvae in a similar fashion to humans (Tsedensodnom et al., 2013). The ethanol-injured larvae can survive for several days without external nutrients (Yin et al., 2012), thus their liver damage is not related to changes in nutrient metabolism. The rapid external development and translucence of the larvae and the availability of fluorescence reporter lines labeling different hepatic cell types make it easy to characterize action of alcohol at cellular resolution. Intriguingly, exposing 4-day-old larvae to 2% ethanol for 24 h is sufficient to induce hepatic steatosis and HSC activation (Passeri et al., 2009; Yin et al., 2012). The larvae acute alcoholic liver injury model reveals the immediate responses of different hepatic cell types to alcohol that likely occur in binge drinking. It also provides insights into the pathogenesis of chronic alcoholic liver injury.

In this study, we use the zebrafish model to demonstrate that blockade of VEGFR activity post-acute ethanol treatment enhances liver repair by ameliorating hepatic steatosis, angiogenesis and fibrogenesis. HSCs and endothelial cells, but not hepatic parenchymal cells, exhibit robust changes in the expression of VEGF receptor genes upon acute ethanol exposure and are likely the direct targets of VEGFR inhibition. By conducting ethanol treatment experiments on *cloche* (also known as *npas4l*) mutants lacking hepatic endothelial cells (Reischauer et al., 2016), we revealed that the effect of VEGFR inhibition on hepatic steatosis and fibrogenesis could be uncoupled from angiogenesis.

## RESULTS

### Inhibition of VEGF receptor tyrosine kinase blocked the increase of HSC number after acute ethanol treatment

**Fig. 1.**
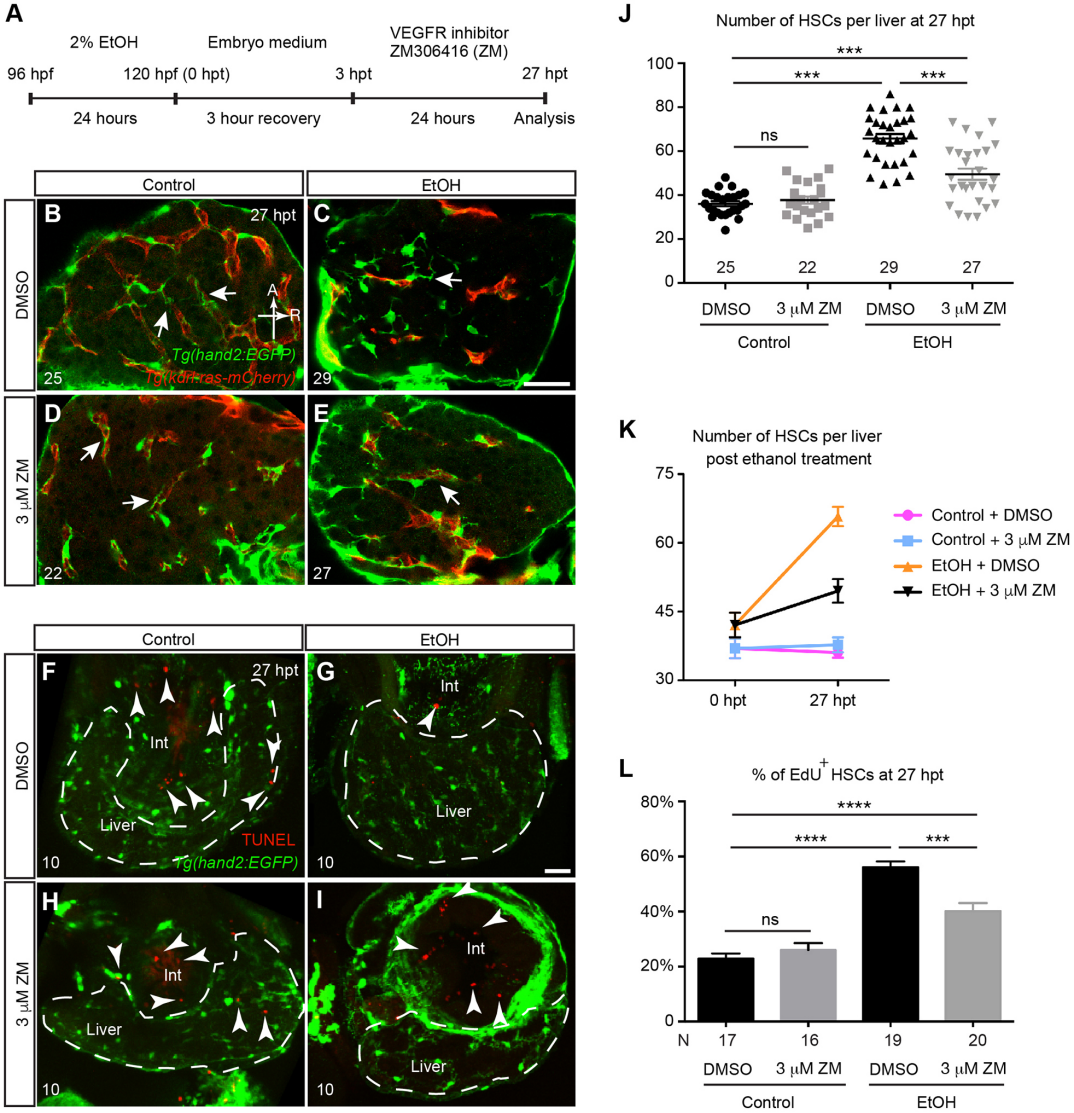
**VEGFR inhibition suppressed the increase of HSC cell number post-acute ethanol exposure.** (A) Work flow of the ethanol and VEGF receptor (VEGFR) inhibitor treatment. (B-E) Representative confocal single-plane images of *Tg(hand2:EGFP;kdrl:ras-mCherry)* larvae treated with 0.03% DMSO (B), 2% ethanol followed by DMSO (C), 3 μM VEGFR inhibitor ZM (D), and 2% ethanol followed by 3 μM ZM (E) at 27 h post-ethanol treatment (hpt). The HSCs are labeled by *Tg(hand2:EGFP)* expression (green), and the endothelial cells are marked by *Tg(kdrl:ras-mCherry)* expression (red). Arrows point to the cellular processes of HSCs. Ventral view, anterior is to the top. Sample sizes are indicated. A, anterior; R, right. Scale bar: 50 μm. (F-I) Confocal three-dimensional projections of vibratome transverse sections stained by TUNEL assay. The apoptotic cells are shown in red (arrowheads), and the HSCs are labeled by *Tg(hand2:EGFP)* expression (green). Very few apoptotic cells were present in the livers of all the control and experimental groups, but were frequently seen in the intestine (int). For each group, three liver sections per larva from 10 larvae were analyzed. Dashed line marks the liver. Scale bar: 30 μm. (J) Numbers (mean±s.e.m.) of HSCs per liver in each treatment group at 27 hpt. (K) Changes in the numbers (mean±s.e.m.) of HSCs per liver in each treatment group between 0 and 27 hpt. The numbers of animals analyzed are indicated in J and Fig. S1F. (L) Percentages (mean±s.e.m.) of HSCs that incorporated EdU labeling in each treatment group. (J-L) Each experiment was repeated at least three times and the numbers of animals analyzed are shown. Statistical significance in J was calculated by one-way ANOVA and Tukey's post-hoc test; in L it was determined by two-tailed Student's *t*-test. ***P<0.001; ****P<0.0001; ns, not significant.

Expression of VEGF ligands and receptors is elevated in fibrotic livers (reviewed by Elpek, 2015) and inhibition of VEGF signaling suppresses activation of mammalian HSCs both *in vivo* and *in vitro* (Liu et al., 2009). We previously showed that inhibition of VEGF signaling significantly reduced the number of HSCs formed during liver development in zebrafish (Yin et al., 2012). To test whether VEGF signaling mediates the responses of HSCs to acute alcohol insults, we treated zebrafish larvae with 2% ethanol from 96 to 120 h post-fertilization (hpf) (Passeri et al., 2009), and used 3 μM ZM306416 hydrochloride (ZM), a chemical inhibitor of Kdr and Flt1 tyrosine kinases, to block VEGF signaling (Hennequin et al., 1999). We used *Tg(hand2:EGFP;kdrl:ras-mCherry)* fish, in which the HSCs and endothelial cells are marked by *Tg(hand2:EGFP)* and *Tg(kdrl:ras-mCherry)* expression, respectively (Yin et al., 2012). We conducted two sets of experiments either by co-treatment of ZM and ethanol for 24 h (Fig. S1A), or by adding ZM after ethanol exposure (Fig. 1A). The acute ethanol treatment caused pericardial edema, lordosis and erratic swimming behaviors in the zebrafish larvae (Passeri et al., 2009). We found that co-treatment of ZM and ethanol as well as adding ZM immediately after ethanol exposure worsened these phenotypes and led to high mortality (data not shown). In contrast, keeping the larvae in embryo medium for 3 h in between 24 h of ethanol treatment and 24 h of ZM treatment improved the survival rate to nearly 100% and the body phenotypes were recovered.

In wild-type (WT) uninjured control livers treated with DMSO, the HSCs extended long cytoplasmic processes to wrap around the hepatic blood vessels (Fig. 1B, arrows). ZM treatment alone did not cause obvious changes in HSC morphology (Fig. 1D, arrows). The HSCs in the ethanol-injured livers had shorter cellular processes (Fig. 1C, arrow) (Yin et al., 2012). In the livers that were co-treated with ethanol and ZM, the HSCs had altered morphology that was similar to those treated by ethanol alone (Fig. S1C,E). In contrast, when ZM was added from 3 to 27 h post-ethanol treatment (hpt), the HSCs resumed the cellular processes and exhibited similar morphology as the uninjured controls (Fig. 1B,E, arrows).

We counted the number of HSCs after ethanol treatment. At 0 hpt, the ethanol-injured larvae had comparable numbers of HSCs as the uninjured control, and co-treatment with ethanol and ZM did not change HSC number (Fig. S1F). We detected a dramatic increase of HSC number in the ethanol-injured livers within 27 hpt (from 42±9 to 66±11, mean±s.d.; Fig. 1J,K), whereas HSC number in the uninjured control remained the same during the equivalent time period (from 37±7 to 36±6) (Yin et al., 2012). The increase of HSC number was less evident when the larvae were treated with ZM between 3 and 27 hpt (from 42±9 to 50±13; Fig. 1J,K). ZM treatment did not change HSC number in the uninjured control (Fig. 1J,K).

Taken together, these results show that inhibition of VEGF signaling after acute ethanol treatment suppresses the changes in HSC morphology and the increase in HSC number.

### Inhibition of VEGF signaling decreased HSC proliferation

In zebrafish larvae, the increase of HSC number after acute ethanol treatment is at least partially due to proliferation of existing HSCs (Yin et al., 2012). It has been reported that inhibition of VEGF signaling induces cell cycle arrests and apoptosis in mammalian HSCs *in vitro* (Liu et al., 2009). We performed a TUNEL assay and detected very few apoptotic cells in the livers of all the experimental groups at 27 hpt (Fig. 1F-I). Therefore, inhibition of VEGF signaling does not seem to induce HSC apoptosis in acute alcoholic liver injury in zebrafish larvae.

To determine whether VEGFR inhibition suppressed HSC proliferation after acute ethanol treatment, we examined incorporation of proliferation marker 5-ethynyl-2′-deoxyuridine (EdU) by the HSCs at 27 hpt. In the livers treated with DMSO only, 23% of the HSCs incorporated EdU (Fig. 1L). In the ethanol-injured livers, the percentage of EdU-positive HSCs increased to 56%. Addition of ZM reduced the percentage of EdU-positive HSCs to 40% in the ethanol-injured animals, but had no effect on the uninjured controls. Thus, inhibition of VEGF signaling post-ethanol treatment suppressed HSC proliferation.

### Inhibition of VEGF signaling decreased extracellular matrix protein deposition and fibrogenic gene expression that were induced by acute ethanol treatment

**Fig. 2.**
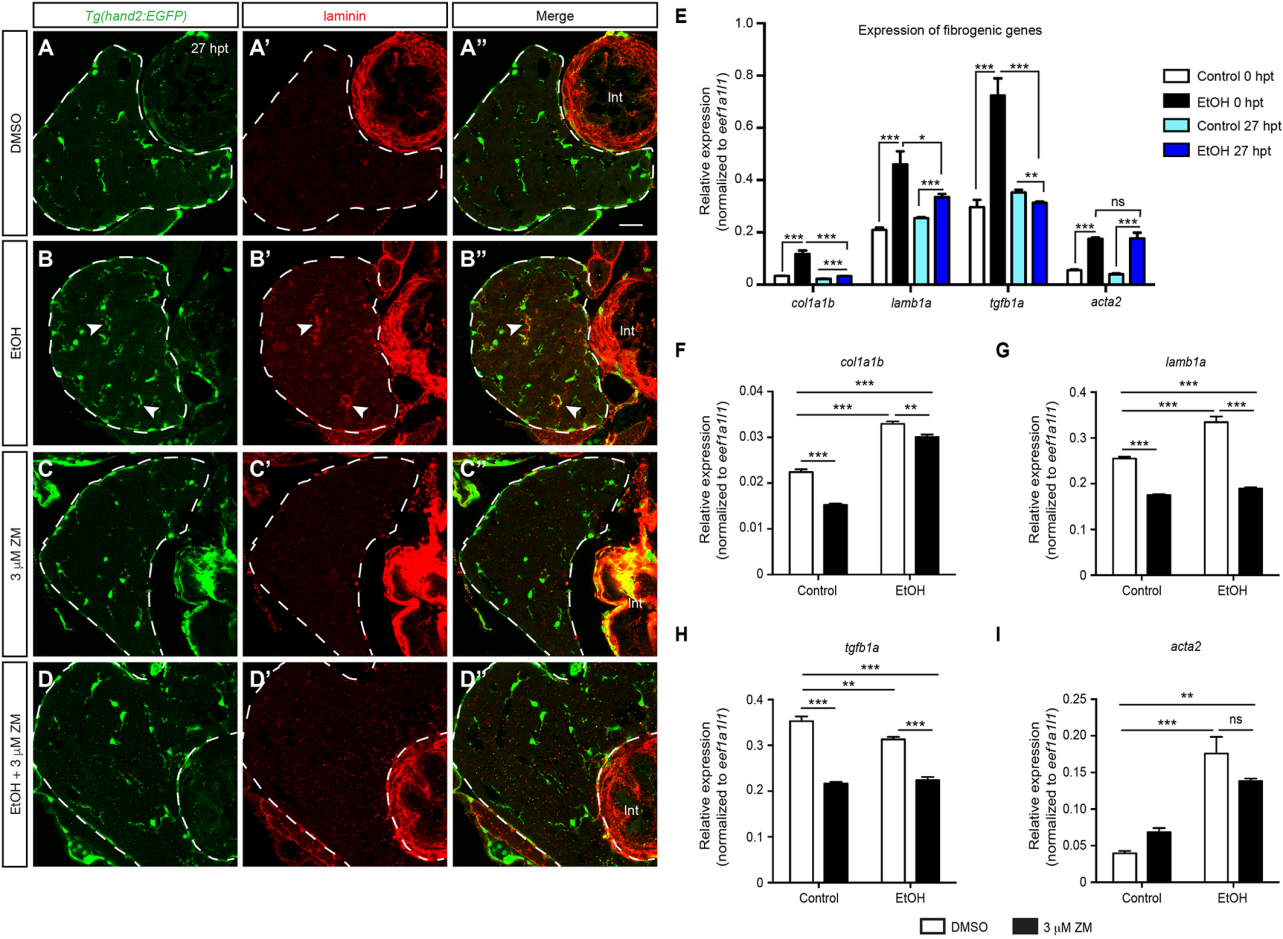
**VEGFR inhibition attenuated fibrogenic responses post-acute ethanol treatment.** (A-D″) Confocal single-plane images from the vibratome transverse sections showing that ZM treatment after acute ethanol exposure suppressed laminin deposition. (A-D) HSCs marked by *Tg(hand2:EGFP)* expression; (A′-D′) laminin deposition; and (A″-D″) merged images of the two. Dashed line marks the liver. Scale bar: 20 μm. Ten larvae were examined in each experimental group. Arrowheads in B-B″ point to the HSCs that secreted laminin. Int, intestine. (E) qPCR analyses showing the comparison of *col1a1b*, *lamb1a*, *tgfb1a*, and *acta2* expression in control and ethanol-treated livers at 0 and 27 hpt. (F-I) qPCR analyses showing the comparison of *col1a1b* (F), *lamb1a* (G), *tgfb1a* (H), and *acta2* (I) expression in the livers of larvae treated with DMSO, ZM, ethanol followed by DMSO, and ethanol followed by ZM at 27 hpt. (E-I) Triplicates were performed. The results are represented as relative expression levels that are normalized to the housekeeping gene *eef1a1l1* (mean±s.e.m.). Statistical significance was calculated by one-way ANOVA and Tukey's post-hoc test. *P<0.05; **P<0.01; ***P<0.001; ns, not significant.

Acute ethanol treatment also induces fibrogenic responses in the larval livers as evident by the deposition of extracellular matrix proteins (Yin et al., 2012). Whereas laminin was almost undetectable in the livers of the uninjured control larvae and the larvae treated with ZM alone (Fig. 2A′,C′), its deposition was drastically increased in the ethanol-injured livers (Fig. 2B′). Laminin deposition was still seen in the larvae that were co-treated with ethanol and ZM (Fig. S2D′). Strikingly, when we added ZM after ethanol treatment, laminin deposition became largely diminished at 27 hpt (Fig. 2D′).

We detected continuous activation of HSCs after ethanol removal as shown by HSC proliferation and laminin deposition, which was suppressed by ZM treatment. These observations raised the question whether inhibition of VEGF signaling enhanced the liver repair or prevented the liver from developing further damage after ethanol treatment. To address this question, we decided to compare the expression levels of fibrogenic genes at 0 and 27 hpt. We isolated the livers from the control and ethanol-injured larvae at 0 and 27 hpt, and performed real-time quantitative PCR (qPCR). We detected elevated expression of *actin, alpha 2, smooth muscle aorta* (*acta2*; also known as *alpha-SMA*); *collagen, type I, alpha 1b* (*col1a1b*); *laminin, beta 1a* (*lamb1a*); *lysyl oxidase* (*lox*); *transforming growth factor, beta 1a* (*tgfb1a*); *thrombospondin 1a* (*thbs1a*) and *TIMP metallopeptidase inhibitor 2b* (*timp2b*) in the ethanol-injured livers at 0 hpt (Fig. 2E and data not shown), all of which have been reported to be elevated in mammalian HSCs during fibrosis progression and/or culture activation (Liu et al., 2004; Takahara et al., 2006). At 27 hpt, the expression of *col1a1b*, *lamb1a* and *acta2*, but not *tgfb1a* remained elevated in the ethanol-treated livers compared with the untreated control (Fig. 2E). In contrast, the expression of *col1a1b*, *lamb1a* and *tgfb1a* was significantly lower than those detected at 0 hpt, indicating that the liver was undergoing self-repair at 27 hpt. The increase of fibrogenic gene expression was suppressed by ZM treatment post-ethanol exposure (Fig. 2F-I). Together, inhibition of VEGF signaling facilitates liver repair after ethanol removal by attenuating fibrogenic responses.

### Inhibition of VEGF signaling impeded hepatic angiogenesis after acute ethanol exposure

**Fig. 3.**
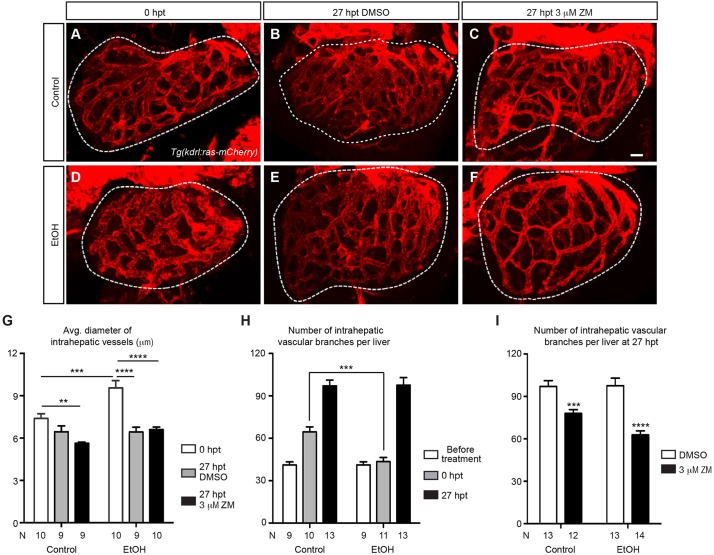
**VEGFR inhibition suppressed hepatic angiogenesis that intensified after acute ethanol treatment.** (A-F) Confocal three-dimensional projections of the whole liver in the control and ethanol-injured larvae immediately after ethanol treatment (0 hpt) (A,D), and after 3 h of recovery period plus 24 h of subsequent DMSO or ZM treatment (27 hpt) (B,C,E,F). The intrahepatic vasculature is marked by *Tg(kdrl:ras-mCherry)* expression. Ventral view, anterior is to the top. Dashed line outlines the liver. Scale bar: 30 μm. (G) Average diameter of intrahepatic vessels (mean±s.e.m.) in control and ethanol-treated livers with or without subsequent ZM treatment. (H) Numbers (mean±s.e.m.) of intrahepatic vascular branches per liver before ethanol treatment, immediately after ethanol treatment (0 hpt) and at 27 h after ethanol treatment (27 hpt). (I) Numbers (mean±s.e.m.) of intrahepatic vascular branches per liver in the larvae treated with DMSO alone, ZM alone, ethanol followed by DMSO, and ethanol followed by ZM, at 27 hpt. (G-I) Each experiment was repeated three times and the numbers of animals analyzed are shown. Statistical significance in G was calculated by one-way ANOVA and Tukey's post-hoc test, and in H,I by two-tailed Student's *t*-test. **P<0.01; ***P<0.001; ****P<0.0001.

VEGF is a potent pro-angiogenic factor, and angiogenesis contributes to the progression of many forms of chronic liver diseases (reviewed by Fernandez et al., 2009). We decided to investigate how the hepatic endothelial cells responded to acute ethanol treatment in zebrafish larvae and whether blocking VEGF signaling affected such responses. As marked by *Tg(kdrl:ras-mCherry)* expression, the intrahepatic blood vessels were dilated at 0 hpt (Fig. 3A,D,G). We assessed hepatic angiogenesis by counting the number of vascular branches. In the uninjured control, the average number of vascular branches per liver increased from 41 to 65 between 96 and 120 hpf (Fig. 3H), in accordance with the growth of the liver during this period. Acute ethanol treatment did not seem to perturb liver growth during this period (data not shown). However, the number of vascular branches did not increase over the duration of ethanol exposure (Fig. 3H), indicating that the development of the hepatic vasculature was stalled in the presence of ethanol.

After ethanol was removed, the blood vessels in the ethanol-injured livers became less dilated at 27 hpt (Fig. 3B,E,G). Between 0 and 27 hpt, we observed a bigger increase in vascular branch number in the ethanol-injured animals (from 41 to 98 branches per liver) compared with the uninjured controls (from 65 to 97 branches per liver) (Fig. 3A,B,D,E,H). Therefore, hepatic angiogenesis intensified in the ethanol-injured animals once ethanol was removed. ZM treatment had no impact on vessel diameters with or without prior ethanol exposure (Fig. 3B,C,E-G; *P*>0.35). However, it reduced vascular branch number in both the uninjured control and the ethanol-injured larvae, with the reduction being more evident in the injured larvae (Fig. 3C,F,I). We conclude that VEGFR inhibition suppressed angiogenesis that is associated with both normal liver growth and acute ethanol-induced liver injury.

### Inhibition of VEGF signaling attenuated hepatic steatosis in the ethanol-injured livers

**Fig. 4.**
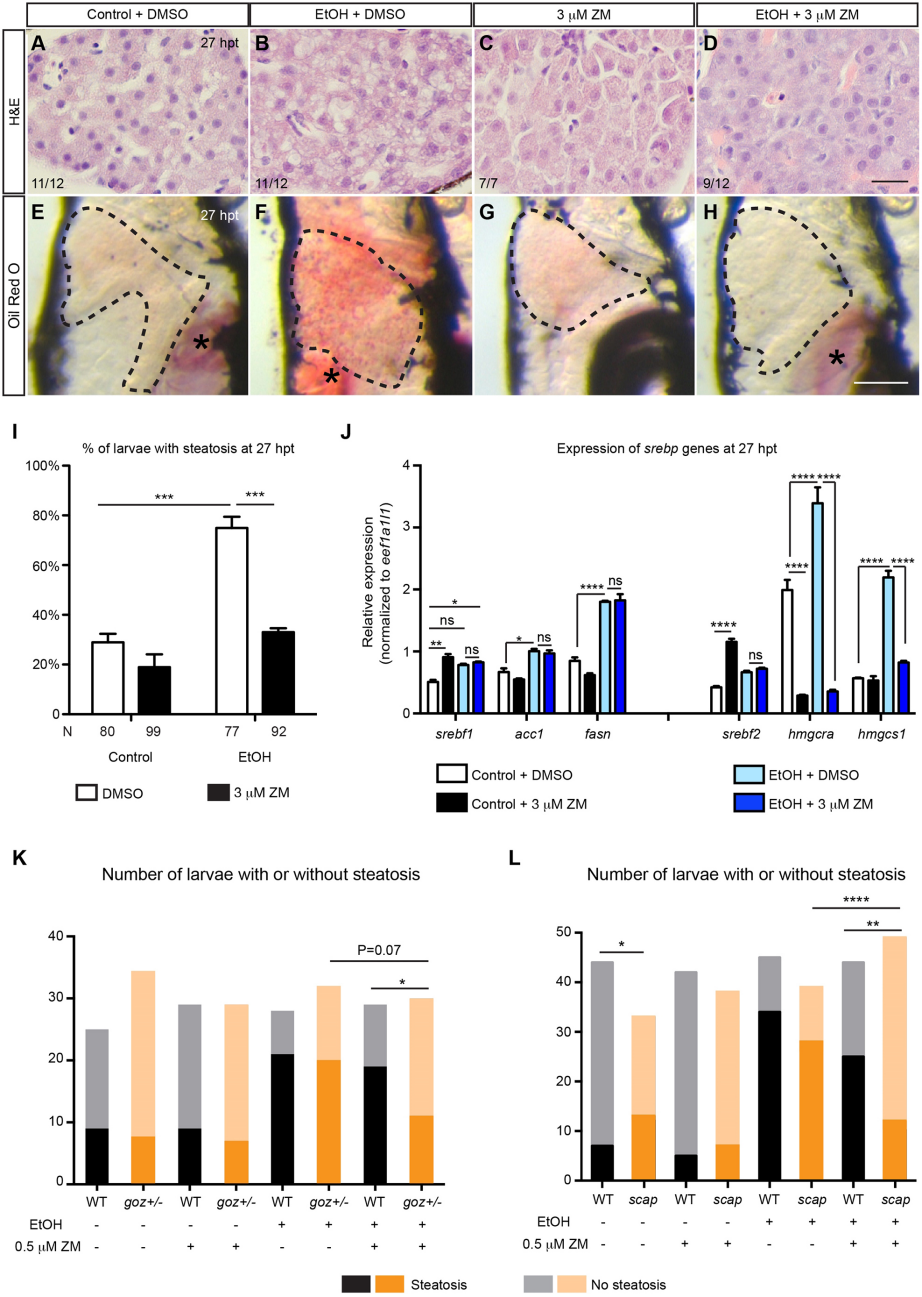
**VEGFR inhibition ameliorated hepatic steatosis post-acute ethanol treatment.** (A-D) Hematoxylin and eosin (H&E) staining of paraffin sections showing livers treated with DMSO alone, ZM alone, ethanol followed by DMSO, and ethanol followed by ZM, at 27 hpt. The total numbers of livers examined and the numbers of livers that exhibited the representative phenotypes are indicated. Scale bar: 20 μm. (E-H) Representative images of the whole-mount Oil Red O staining in different experimental groups. Dashed line marks the liver. Lateral view, anterior is to the top. Oil Red O also stains the residual yolk tissue and swim bladder (asterisks). Scale bar: 250 μm. (I) Percentages (mean±s.e.m.) of the larvae with hepatic steatosis in different experimental groups at 27 hpt based on Oil Red O staining. The experiments were repeated four times and the number of animals analyzed is listed in each column. (J) qPCR analyses showing the hepatic expression of *srebf1* genes (left) and *srebf2* genes (right) in different experimental groups at 27 hpt. Triplicates were performed. The results are represented as relative expression levels that are normalized to the housekeeping gene *eef1a1l1* (mean±s.e.m.). (K) The numbers of WT or *goz^+/−^* mutant larvae that developed steatosis at 27 hpt with or without being treated with 0.5 μM ZM. (L) The numbers of control or *scap* morpholino-injected larvae that developed steatosis at 27 hpt with or without being treated with 0.5 μM ZM. Statistical significance in I was calculated by two-tailed Student's *t*-test, and in J by one-way ANOVA and Tukey's post-hoc test. In K,L analysis of differences in the distribution of phenotypes was performed by the contingency table Fisher's exact test. *P<0.05; **P<0.01; ***P<0.001; ****P<0.0001; ns, not significant.

Similar to mammals, zebrafish larvae develop hepatic steatosis in response to acute ethanol exposure as revealed by both histology and whole-mount Oil Red O staining (Fig. 4B,F,I) (Passeri et al., 2009). We found that treatment with ZM post-ethanol exposure ameliorated hepatic steatosis in zebrafish larvae (Fig. 4D,H,I).

We performed qPCR experiments to examine whether treatment with ZM altered the expression of hepatic function genes in the ethanol-injured livers. Accompanied by hepatic steatosis, the expression of acute phase response genes and genes encoding thioredoxins in the redox signaling was elevated in the ethanol-injured livers (Fig. S3A) (Howarth et al., 2013). Adding ZM post-ethanol exposure further upregulated the expression of these genes. Whereas ethanol treatment alone had little effect on genes involved in xenobiotic metabolism and the complement pathway (Howarth et al., 2013), treating the ethanol-injured larvae with ZM increased their expression (Passeri et al., 2009). Overall, changes in the expression of hepatic function genes cannot explain the inhibitory effect of ZM treatment on steatosis.

Next, we examined the expression of genes involved in β-oxidation, the TNFα signaling pathway and hypoxia, all of which have been associated with alcohol-induced hepatic steatosis (Louvet and Mathurin, 2015). We did not detect any consistent impact of ZM treatment on the expression of these genes (Fig. S3B). Activation of the unfolded protein response (UPR) and endoplasmic reticulum stress also contribute to ethanol-induced steatosis in mammals and zebrafish (Ji, 2012; Ji and Kaplowitz, 2003; Malhi and Kaufman, 2011; Tsedensodnom et al., 2013). Accordingly, we found that the expression of UPR sensor and target genes were increased in ethanol-injured livers at 0 hpt, and their expression was further upregulated when co-treated with ZM (Fig. S4A). ZM treatment post-ethanol exposure did not cause a uniform effect on the expression of UPR sensor and target genes (Fig. S4B). We conclude that the suppression of hepatic steatosis by ZM treatment is not due to decreased UPR activation.

Activation of sterol regulatory element-binding protein (Srebp; also known as Srebf) transcription factors promotes hepatic triglyceride and cholesterol synthesis, leading to steatosis in ALD (Ji et al., 2006). Srebf1 target genes have been implicated in fatty acid synthesis, and Srebf2 target genes are involved in cholesterol synthesis (Ye and DeBose-Boyd, 2011). By qPCR, we found that the expression levels of *srebf1*, *srebf2* and their target genes were significantly increased in ethanol-injured livers compared with the uninjured controls at 27 hpt (Fig. 4J) (Passeri et al., 2009). ZM treatment alone increased the expression of *srebf1* and *srebf2*; however, it did not cause steatosis, likely due to the lack of substrate. Interestingly, adding ZM after ethanol exposure specifically blocked the expression of the Srebf2 target genes *hmgcra* and *hmgcs1*, but not the Srebf1 target genes *acc1* and *fasn* (Fig. 4J).

The qPCR results raised the possibility that VEGFR inhibition might interfere with the Srebp pathway. We tested whether Kdrl acted downstream of the Srebp pathway by treating animals overexpressing the truncated Srebf1 or Srebf2 protein, which terminates before the first transmembrane segment, with ZM (Fig. S5). These proteins enter the nucleus directly without a requirement for proteolysis, and thus are immune to the normal process of downregulation (Shimano et al., 1997). Overexpression of either truncated Srebf1 or Srebf2 protein induced lipid accumulation in the hepatocytes in control WT treated with DMSO. ZM treatment did not abolish the lipid droplets, arguing against the possibility that Kdrl acted downstream of Srebps. In zebrafish, Srebp activation is blocked in embryos injected with a morpholino targeting the *SREBF chaperone* (*scap*) gene, or in a mutant line *gonzo* (*goz*^+/−^) with a mutation in the *membrane-bound transcription factor protease 1* (*mbtps1*) gene (Passeri et al., 2009). Whereas treatment with 0.5 μM ZM did not attenuate ethanol-induced steatosis in WT, the same dose blocked steatosis in *goz*^+/−^ embryos (Fig. 4K). The low dose of ZM also blocked steatosis in embryos injected with 0.5 pmol *scap* morpholino, a dose that did not suppress steatosis on its own (Fig. 4L). These findings suggest that VEGF signaling and Srebps interact to contribute to the induction of hepatic steatosis by acute ethanol treatment.

### *kdrl* mutants showed similar hepatic responses to acute ethanol treatment as the VEGFR inhibitor-treated larvae

**Fig. 5.**
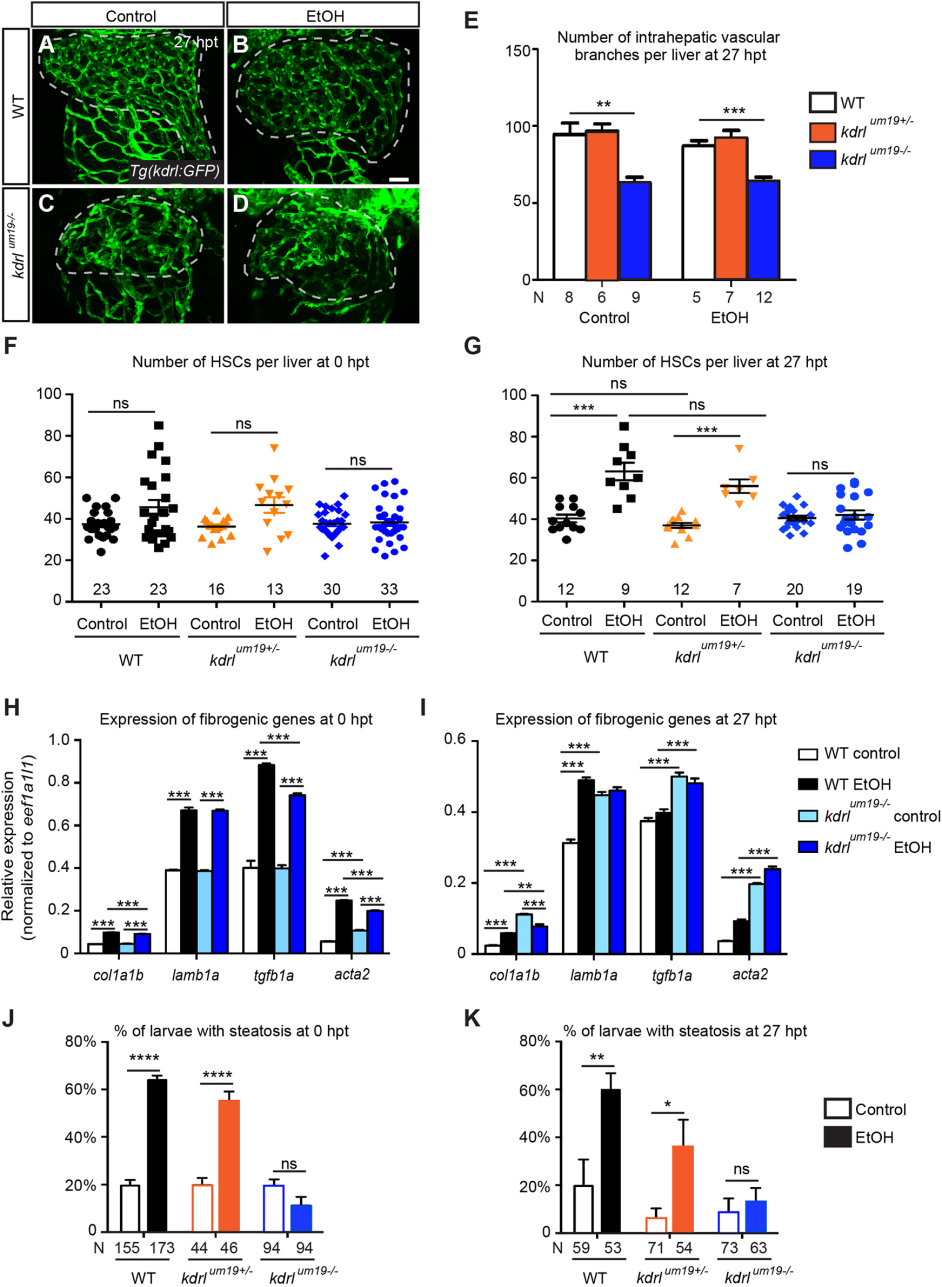
***kdrl* mutants developed similar hepatic injuries after acute ethanol treatment as the ZM-treated WT larvae.** (A-D) Confocal three-dimensional projections showing the whole-liver intrahepatic vasculature in WT and *kdrl^um19^* mutant larvae at 27 hpt. The intrahepatic vasculature is marked by *Tg(kdrl:GFP)* expression. Ventral view, anterior is to the top. Dashed line marks the liver. Scale bar: 40 μm. (E) Numbers (mean±s.e.m.) of intrahepatic vascular branches per liver in WT, *kdrl* heterozygotes and homozygous mutants at 27 hpt. (F,G) Numbers (mean±s.e.m.) of HSCs in WT, heterozygotes and mutants in different experimental groups at 0 hpt (F) and 27 hpt (G). (H,I) qPCR analyses showing the comparison of *col1a1b*, *lamb1a*, *tgfb1a* and *acta2* expression in control and ethanol-treated WT and mutant livers at 0 and 27 hpt. Triplicates were performed. The results are represented as relative expression levels normalized to the housekeeping gene *eef1a1l1* (mean±s.e.m.). (J,K) Percentages (mean±s.e.m.) of WT (black), *kdrl* heterozygotes (orange) and homozygous mutants (blue) with hepatic steatosis at 0 hpt (J) and 27 hpt (K) based on Oil Red O staining. Each experiment in E-G was repeated three times and the numbers of larvae analyzed are shown. Statistical significance in E,J,K was calculated by two-tailed Student's *t*-test, and in F-I by one-way ANOVA and Tukey's post-hoc test. *P<0.05; **P<0.01; ***P<0.001; ****P<0.0001; ns, not significant.

One caveat of using ZM is that it acts on multiple VEGFRs and might target other signaling pathways involved in the pathophysiology of acute ethanol-induced liver injury (Hennequin et al., 1999). To address this issue, we performed acute ethanol treatment on a *kdrl^um19^* mutant that carries a 4 bp deletion in exon 2 of the *kdrl* gene (Meng et al., 2008). The deletion truncates the Kdrl receptor tyrosine kinase in the extracellular domain and is likely a null allele. Unlike *Kdr* mutant mice that die during early development due to a severe reduction of all blood vessels (Shalaby et al., 1995), zebrafish *kdrl^um19^* mutants exhibit milder vascular phenotypes (Covassin et al., 2006). Some mutants survived to 7 days post-fertilization (dpf) and their liver sizes were comparable with WT (Fig. 5A,C; data not shown). We treated *kdrl^um19^* mutants and their siblings with 2% ethanol from 96 to 120 hpf, removed the ethanol, and analyzed the liver injury at 0 and 27 hpt. Expression of the *Tg(kdrl:GFP)* transgene (Jin et al., 2005) revealed that in both uninjured control and ethanol-injured groups, *kdrl^um19^* mutants had fewer hepatic vascular branches than the WT and heterozygous siblings at 27 hpt (Fig. 5A-E), indicative of impaired angiogenesis.

Without ethanol, *kdrl^um19^* mutants had similar number of HSCs as WT and heterozygotes at both 0 and 27 hpt (Fig. 5F,G). Ethanol treatment did not change HSC number at 0 hpt in all animals (Fig. 5F). At 27 hpt, HSC number was significantly increased in ethanol-injured WT and heterozygous larvae, but not in the mutants (Fig. 5G). In terms of fibrogenesis, the uninjured control WT and *kdrl^um19^* mutant livers showed similar baseline expression of fibrogenic genes at 0 hpt (Fig. 5H). Both ethanol-treated WT and mutant livers exhibited increased expression of fibrogenic genes right after ethanol exposure. Thus, the mutant liver developed a fibrogenic response to ethanol. At 27 hpt, the uninjured control mutant livers had higher fibrogenic gene expression than the control WT (Fig. 5I). We reasoned that the liver was undergoing rapid growth at this stage, which might trigger liver damage in the mutant owing to impaired angiogenesis. Expression of fibrogenic genes remained elevated in ethanol-treated WT, but not in the mutant at 27 hpt. Taken together, these results show that the mutant liver exhibited fibrogenesis in the presence of ethanol, but rapidly diminished fibrogenic responses after ethanol was removed.

At both 0 and 27 hpt, *kdrl^um19^* mutants were protected from developing ethanol-induced hepatic steatosis as revealed by Oil Red O staining (Fig. 5J,K). We examined the expression of UPR sensor and target genes in WT and mutants at 0 and 27 hpt by qPCR (Fig. S4C,D) and found that ethanol-treated *kdrl^um19^* mutants maintained high levels of UPR gene expression at both stages. This result confirms that VEGF signaling does not regulate hepatic steatosis through affecting the UPR.

Taken together, we conclude that during ethanol exposure, ethanol and its metabolites likely have a direct impact on fibrogenic gene expression independent of VEGF signaling. After ethanol is removed, VEGF signaling maintains the elevated expression of fibrogenic genes in the injured livers and promotes HSC proliferation. Secondly, VEGF signaling is required for the development of steatosis during and after ethanol treatment.

### The cell source of VEGF ligands and receptors after acute ethanol treatment

**Fig. 6.**
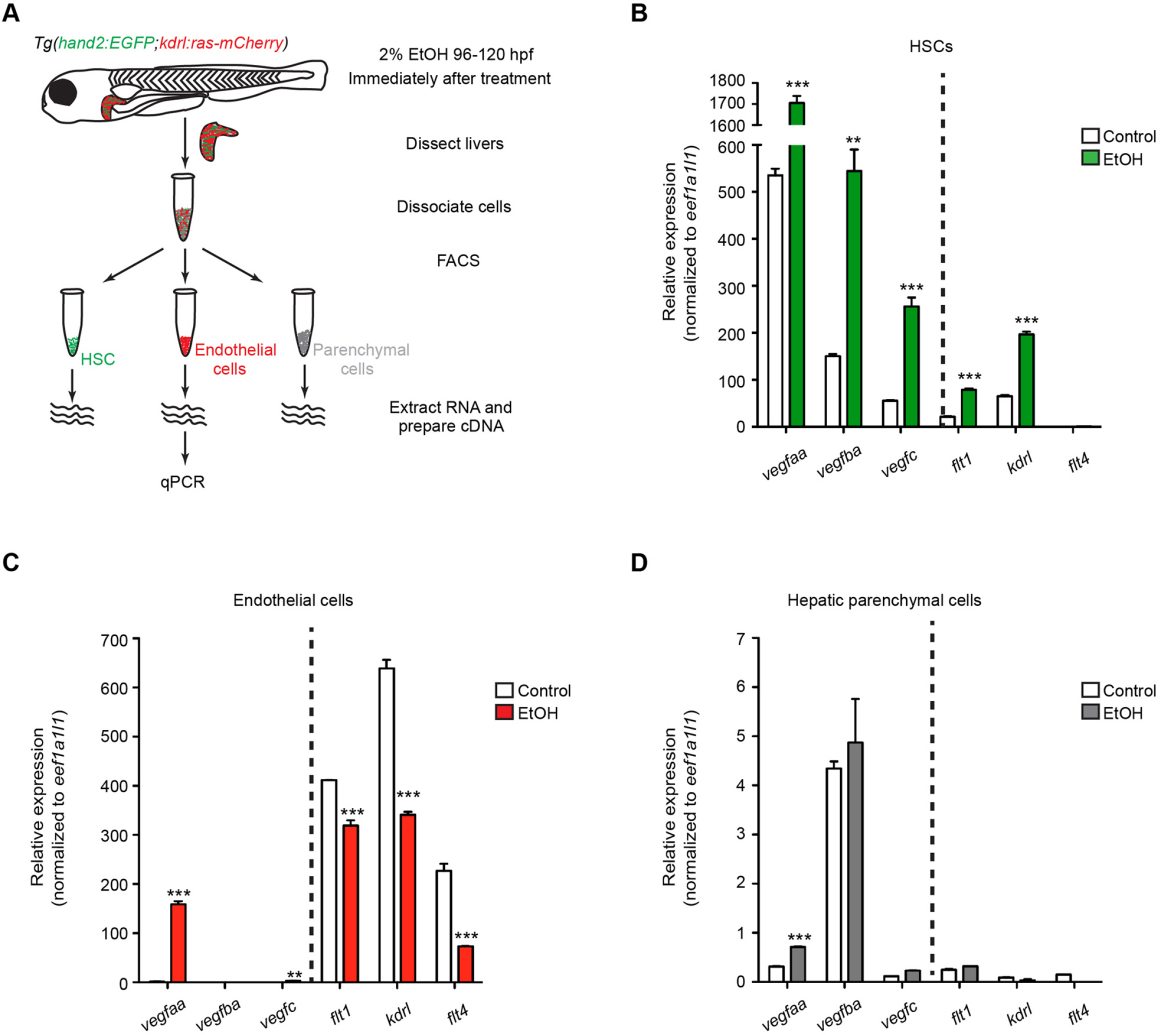
**Expression of VEGF ligand and receptor genes in different hepatic cell types at immediately after acute ethanol treatment.** (A) Work-flow of FACS and qPCR experiment. (B-D) Expression levels of VEGF ligand and receptor genes in control and ethanol-treated HSCs (B), endothelial cells (C) and hepatic parenchymal cells (D). Triplicates were performed for each condition. The results are represented as relative expression levels normalized to the housekeeping gene *eef1a1l1* (mean±s.e.m.). Statistical significance was calculated by one-way ANOVA and Tukey's post-hoc test. **P<0.01; ***P<0.001.

To understand how VEGF signaling regulates the responses of different liver cell types to acute alcohol insults, we performed qPCR to examine the expression of VEGF ligand and receptor genes in the HSCs, endothelial cells and liver parenchymal cells immediately after ethanol treatment (Fig. 6A). We isolated the *Tg(hand2:EGFP)*-expressing HSCs and *Tg(kdrl:ras-mCherry)*-expressing endothelial cells by fluorescence-activated cell sorting (FACS). The remaining cells that did not express either transgene should mostly be hepatocytes and cholangiocytes. Although qPCR detected expression of *kdrl* in the HSCs, we did not observe co-localization of *Tg(hand2:EGFP)* and *Tg(kdrl:ras-mCherry)* expression in either the control or ethanol-treated livers (Fig. 1B,C). We detected expression of HSC marker *foxf1* (Kalinichenko et al., 2003) only in the *Tg(hand2:EGFP)*-expressing cells, and enrichment of endothelial cell marker *cdh5* (also known as *VE-cadherin*) (Larson et al., 2004) only in the *Tg(kdrl:ras-mCherry)*-expressing cells (Fig. S6B,C), confirming the purity of the isolated HSCs, endothelial cells and parenchymal cells. We also excluded the possibility that there was contamination of the HSC samples with endothelial progenitors that did not express *cdh5* (Fig. S6D-G″).

Among all the cell types examined, the HSCs exhibited the most robust increase in the expression of VEGF ligand and receptor genes (Fig. 6B). In the control larvae, the HSCs expressed VEGF ligand genes *vegfaa*, *vegfba* and *vegfc*. Their expression was increased by more than threefold immediately after ethanol treatment. For the receptors, the expression levels of *flt1* and *kdrl* were significantly increased in the HSCs after ethanol treatment. In the endothelial cells, the expression of *vegfaa* was induced after ethanol exposure (Fig. 6C). The control and ethanol-treated endothelial cells expressed all three VEGF receptor genes, but their expression was lower in the ethanol-treated endothelial cells, consistent with the observation that angiogenesis was stalled during ethanol treatment (Fig. 3G). The hepatic parenchymal cells expressed VEGF ligand genes, but there was no significant difference between the uninjured control and the ethanol-injured cells except for *vegfaa* (Fig. 6D). Both the control and ethanol-injured parenchymal cells only showed minimal expression of VEGF receptor genes.

Immune cells can also serve as the source of VEGF signaling in organ injury (reviewed by Bruno et al., 2014). We used *Tg(lyz:GFP)* (Hall et al., 2007) and *Tg(mpeg1:YFP)* (Roca and Ramakrishnan, 2013) transgenic fish to visualize neutrophils and macrophages, respectively, after acute ethanol exposure. We only detected an average of one or two neutrophils in the livers of the ethanol-injured larvae at 0 and 27 hpt (Fig. S5A-E). Both the uninjured control and ethanol-injured livers had about five macrophages at 0 hpt (Fig. S5J). At 27 hpt, whereas the uninjured control livers only had an average of four macrophages per liver, the ethanol-injured larvae had 10 macrophages per liver (Fig. S5F-J). Nevertheless, given the low numbers of macrophages and neutrophils, they might not be the main sources of VEGFs and their receptors in our acute ethanol injury model. We did not investigate the involvement of the adaptive immune system as in zebrafish it is morphologically and functionally mature only 4-6 weeks post-fertilization (Novoa and Figueras, 2012).

In summary, the HSCs are the first liver cell type to increase the expression of VEGF ligand and receptor genes upon acute ethanol treatment. The HSCs and endothelial cells, but not the hepatic parenchymal cells, express *kdrl* and are likely the main cell types that are directly affected by VEGFR inhibition.

### Reduced VEGF signaling in HSCs rather than impaired angiogenesis caused by ZM treatment attenuated ethanol-induced hepatic steatosis

**Fig. 7.**
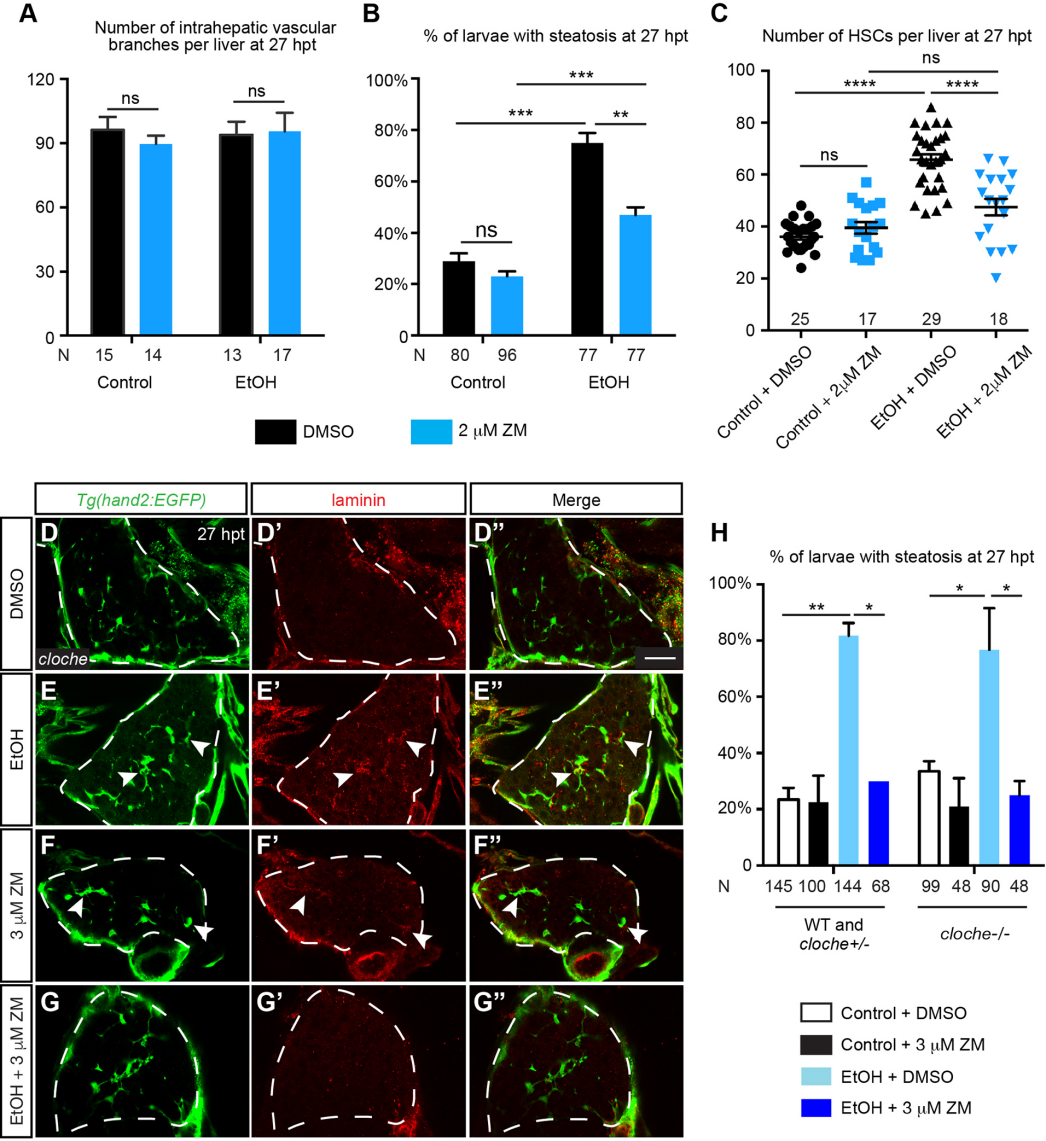
**Ethanol-induced fibrogenesis and steatosis could be uncoupled from angiogenesis.** (A) Numbers (mean±s.e.m.) of intrahepatic vascular branches per liver at 27 hpt in uninjured WT larvae treated with DMSO, uninjured larvae treated with 2 μM ZM, ethanol-exposed larvae treated with DMSO, and ethanol-exposed larvae treated with 2 μM ZM. (B) Percentages (mean±s.e.m.) of animals in different experimental groups with hepatic steatosis at 27 hpt based on Oil Red O staining. (C) Numbers (mean±s.e.m.) of HSCs per liver in different experimental groups at 27 hpt. (D-G″) Confocal single-plane images from the vibratome transverse sections showing that ZM treatment after acute ethanol exposure suppressed laminin deposition in *cloche* mutants. (D-G) HSCs marked by *Tg(hand2:EGFP)* expression; (D′-G′) laminin deposition; and (D″-G″) merged images of the two. Ventral views, anterior is to the top. Dashed line marks the liver. Scale bar: 30 μm. Ten larvae were examined in each experimental group. Arrowheads mark the HSCs that secreted laminin. (H) Percentages (mean±s.e.m.) of WT plus *cloche* heterozygotes, and homozygous *cloche* mutants treated with ethanol followed by ZM that exhibited hepatic steatosis at 27 hpt based on Oil Red O staining. Statistical significance in A,B,H was calculated by two-tailed Student's *t*-test, and in C one-way ANOVA and Tukey's post-hoc test. *P<0.05; **P<0.01; ***P<0.001; ****P<0.0001; ns, not significant.

ZM treatment suppressed ethanol-induced hepatic steatosis, fibrogenesis and angiogenesis, but how are they crosslinked? Whereas 3 μM ZM treatment caused a significant reduction of intrahepatic vascular branches (Fig. 3I), 2 μM ZM treatment did not seem to impair angiogenesis in either control or ethanol-injured livers (Fig. 7A). In contrast, 2 μM ZM treatment was sufficient to suppress hepatic steatosis and increase of HSC number at 27 hpt (Fig. 7B,C), suggesting that steatosis and HSC proliferation can be suppressed by VEGF inhibition without impairment of angiogenesis. In a second approach, we performed acute ethanol treatment on *cloche* mutants that carry a null mutation in the *cloche* gene encoding a bHLH-PAS transcription factor (Reischauer et al., 2016). These animals lack most hematopoietic and endothelial cells but form normal numbers of HSCs during liver development (Stainier et al., 1995; Yin et al., 2012). Whereas laminin was almost undetectable in uninjured *cloche* mutants at 27 hpt (Fig. 7D-D″), we observed a drastic increase in laminin deposition in the ethanol-injured mutants (Fig. 7E-E″, arrowheads). Treatment with 3 μM ZM induced modest laminin deposition in the uninjured mutant livers (Fig. 7F-F″), but completely abolished laminin deposition in the ethanol-injured mutant livers (Fig. 7G-G″). By Oil Red O staining, we found that most *cloche* mutants exhibited hepatic steatosis at 27 hpt, which was suppressed by ZM treatment (Fig. 7H). In conclusion, hepatic endothelial cells are not required for sustained HSC activation and hepatic steatosis after ethanol treatment. Given that HSCs are the direct target of ZM treatment, our study also suggests that in the absence of endothelial cells, VEGF signaling mediates interactions between hepatocytes and activated HSCs that cause ethanol-induced steatosis.

## DISCUSSION

Here, we have shown that the zebrafish larval liver develops steatosis, fibrogenesis and angiogenesis after just 24 h of acute ethanol exposure. Both pharmacological and genetic perturbation of Kdrl function facilitated the liver recovery post-ethanol exposure. We revealed that HSCs were the first liver cell type to increase the expression of VEGF ligand and receptor genes upon acute ethanol treatment. Both HSCs and endothelial cells, but not hepatic parenchymal cells, showed robust *kdrl* expression, suggesting that angiogenesis and fibrogenesis were directly affected by Kdrl inhibition. Furthermore, we showed that ethanol-induced fibrogenesis and hepatic steatosis could still occur in the absence of hepatic endothelial cells; thus, VEGF signaling regulates paracrine interactions between activated HSCs and hepatocytes underlying hepatic steatosis.

We showed that VEGF signaling moderated HSC activation after acute ethanol exposure. HSCs were the first hepatic cell type to upregulate VEGF ligand and receptor expression upon ethanol treatment. Because *kdrl* mutants still showed fibrogenesis during ethanol treatment, we conclude that VEGF signaling is not required for the initiation of HSC activation in response to alcohol. Rather, it sustains HSC activation after ethanol removal by maintaining extracellular matrix protein deposition and expression of fibrogenic genes, and by promoting HSC proliferation. It is noteworthy that proliferation of existing HSCs might not be the only mechanism underlying the increase of HSC number after acute ethanol exposure. In rodents, the mesothelial cells at the liver surface enter the liver upon injury and differentiate into myofibroblasts (Li et al., 2013). Lineage-tracing studies in mice suggest that portal fibroblasts, bone marrow-derived fibrocytes and potentially liver epithelial cells can give rise to myofibroblast-like cells (Iwaisako et al., 2014; Wells and Schwabe, 2015; Xie and Diehl, 2013). The origin of the myofibroblast pool in ALD has not yet been determined (Bataller and Gao, 2015). Taking advantage of the accessibility of zebrafish larvae to live imaging and the availability of hepatic reporter strains, we plan to determine the origin of myofibroblasts in the zebrafish acute ALD model and investigate the involvement of VEGF signaling in future experiments.

Kdrl inhibition ameliorated hepatic steatosis, angiogenesis and fibrogenesis after ethanol was removed. Dissecting the link among these three pathogenic processes will require cell-type-specific disruption of Kdrl function. Here, we have shown that both HSCs and endothelial cells expressed *kdrl* upon acute ethanol treatment, suggesting that Kdrl inhibition directly targets these two cell types to suppress angiogenesis and HSC activation. Coulon et al. (2013) reported that blockade of VEGFR2 attenuates hepatic steatosis in two rodent models of non-alcoholic steatohepatitis, both in a preventive and therapeutic setting. In zebrafish larvae, hepatic parenchymal cells expressed minimal levels of VEGF receptor genes regardless of ethanol exposure. Thus, the inhibition of ethanol-induced steatosis by ZM treatment is likely to be secondary to the attenuation of angiogenesis and fibrogenesis. Our study of *cloche* mutants provided direct evidence to show that fibrogenesis and steatosis could be uncoupled from angiogenesis. Therefore, VEGF signaling regulates HSC activation upon ethanol treatment, which in turn causes steatosis in the hepatocytes.

We showed that *kdrl* mutants did not develop hepatic steatosis either at 0 hpt or 27 hpt, suggesting that despite not being expressed by hepatocytes, Kdrl might be an obligatory effector for ethanol-induced hepatic steatosis. In both acute and chronic alcoholic liver injury, activation of Srebp transcription factors increases lipogenesis and is required for the development of steatosis (Ji et al., 2006; Passeri et al., 2009; You et al., 2002). Consistently, our data implicate an interaction of VEGF signaling and Srebps in the regulation of steatosis in the zebrafish model of acute ethanol exposure. VEGF induces activation of Srebps in endothelial cells *in vitro* (Zhou et al., 2004), and expression of both VEGF and Srebp1 are elevated in the diabetic kidney (Sun et al., 2002). These studies support the notion that the regulation of Srebp activation by VEGF signaling is not restricted to the hepatocytes. How VEGF inhibition in HSCs affects Srebp activation in the hepatocytes is not clear. Notably, VEGF inhibition mainly reduced the expression of Srebf2 target genes that are involved in cholesterol synthesis. The expression of Srebf1 target genes that regulate fatty acid synthesis and *de novo* lipogenesis remained elevated after ZM treatment. This result suggests that signaling pathways other than *de novo* lipogenesis are also altered by VEGF inhibition, which contributes to the amelioration of steatosis. We found that VEGF inhibition influenced the expression of a few genes involved in xenobiotic metabolism and the complement pathway. To what degree these two pathways are affected by VEGF inhibition in our model and whether it contributes to the attenuation of hepatic steatosis requires further analyses.

Immune cells have been shown to participate in VEGF signaling in organ injury (reviewed by Bruno et al., 2014). They can directly contribute to alcoholic steatosis, angiogenesis and fibrogenesis by producing pro-inflammatory cytokines such as IL-1β and IL-6 (reviewed by Seo and Jeong, 2016). However, we detected very low numbers of macrophages and neutrophils in the ethanol-injured livers. The adaptive immune system is not yet mature and functional at the larval stage (Novoa and Figueras, 2012). Histological analyses of the ethanol-treated livers also indicated no sign of inflammation or infiltration of inflammatory foci. Thus, whether immune cells have a significant role in the pathophysiology of acute ALD in zebrafish larvae remains questionable.

The beneficial effects of ZM treatment on the zebrafish acute alcoholic liver injury model imply that anti-VEGF agents might be candidates for ALD treatment. However, we also showed that blockade of VEGF signaling did not prevent HSC activation and fibrogenesis in the presence of ethanol, suggesting that ethanol and its metabolites directly act on HSCs, independent of VEGF signaling. Therefore anti-VEGF agents might not be useful for individuals with an active alcohol addiction. Instead, our work implies that anti-VEGF agents might enhance liver recovery from alcohol-induced injury upon cessation of alcohol consumption by attenuating angiogenesis, steatosis and HSC activation.

Our work provides a proof-of-concept for using zebrafish to identify molecular mechanisms leading to alcoholic liver injury. ALD in humans takes years of alcohol consumption, and no existing animal models mimic the entire spectrum of the disease in humans (Louvet and Mathurin, 2015). The zebrafish larval model has limitations as it only triggers the acute effects of alcohol on the liver, and the pathophysiology of the larval liver is different from the adult. It will be necessary to validate the findings in an adult ALD model. A recent study described that adult zebrafish develop steatosis, steatohepatitis and fibrosis upon chronic alcohol exposure (Lin et al., 2015). However, we observed that chronic alcohol exposure resulted in hemorrhage and impairment of feeding behavior in adult fish (Y.C., unpublished observation), making it difficult to determine to what degree the liver injuries are directly induced by alcohol. Nevertheless, it was intriguing that zebrafish larvae readily exhibited steatosis, angiogenesis and fibrogenesis after just 24 h of ethanol exposure. By leveraging this unique feature, we designed straightforward assays to rapidly evaluate liver injuries in zebrafish larvae. Given the small size of the larvae and the ease of collecting thousands of embryos on a weekly basis, one exciting future direction will be to use the same assays in high-throughput screens for genetic mutations and chemical compounds that attenuate hepatic injuries induced by acute ethanol insults. The identified targets will likely benefit the development of novel therapies for ALD.

## MATERIALS AND METHODS

### Zebrafish

Wild type (WT), *cloche^s5+/−^*, *kdrl^um19+/−^*, *gonzo^hi1487+/−^*, *Tg(hand2:EGFP)^pd24^*, *Tg(kdrl:ras-mCherry)^s896^*, *Tg(mpeg1:YFP)^w200^*, *Tg(lyz:EGFP)^nz117^*, *Tg(-2.3etv2:etv2-EGFP)^mw204tg^* and *Tg(kdrl:GFP)^s843^* adults and larvae were maintained under standard conditions (Kimmel et al., 1995) approved by the institutional animal care and use committee of Cincinnati Children's Hospital Medical Center (CCHMC). The genotypes of *kdrl^um19^* and *gonzo^hi1487^* heterozygotes and mutants were determined by the PCR genotyping protocols described previously (Meng et al., 2008; Passeri et al., 2009).

### Morpholino injection

The splice-blocking morpholino targeting *scap* and control morpholino were described previously (Passeri et al., 2009). 1 nl of the 0.5 mM stock was injected into one-cell stage WT embryos.

### Chemical treatment

Acute ethanol (Sigma-Aldrich; St Louis, MO) treatment was conducted as described (Passeri et al., 2009). ZM306416 hydrochloride (2499; Tocris, Minneapolis, MN) was made into 10 mM stock in DMSO and diluted to 3 μM in embryo medium prior to use.

### Immunostaining, TUNEL assay and EdU proliferation analysis

Immunostaining on whole-mount larvae and 140 μm vibratome sections were performed as described (Field et al., 2003; Trinh and Stainier, 2004). We used the following antibodies: chicken anti-GFP (GFP-1020; Aves Labs; Tigard, OR) at 1:1000, polyclonal anti-laminin (L9393; Sigma-Aldrich, St Louis, MO) at 1:100, and goat anti-chicken 488 (A-11039; Life Technologies, Carlsbad, CA) at 1:200. To analyze HSC proliferation during ZM306416 treatment, *Tg(hand2:EGFP)* larvae were exposed to 2% ethanol for 24 h, allowed to recover in embryo medium for 3 h, and incubated in 7 µM EdU along with 3 µM ZM306416 for another 24 h. Proliferating cells were detected using Click-iT EdU Imaging Kit (Life Technologies). The TUNEL reaction was performed on 140 μm vibratome sections using ApopTag^®^ Red In Situ Apoptosis Detection Kit as described by the manufacturer's instructions (EMD Millipore, Billerica, MA). The samples were imaged on a Nikon A1Rsi inverted confocal microscope (Nikon Instruments, Melville, NY) at the Confocal Imaging Core at CCHMC. Image processing and quantification were conducted using Imaris software (Bitplane, Concord, MA).

### qPCR

To determine the expression levels of fibrogenic genes, Srebp genes, hepatic function genes, UPR genes, and genes involved in β-oxidation, hypoxia and the TNFα pathway, total RNAs were prepared from pools of 20 dissected livers per treatment group by using Arcturus PicoPure RNA Isolation Kit (Life Technologies). cDNAs were synthesized by using SuperScript III First-Strand Synthesis System (Life Technologies). 300 ng of each cDNA sample and PCR primers were added to SYBR^®^ Green qPCR Master Mix (Life Technologies). To quantify the expression levels of VEGF ligand and receptor genes in different hepatic cell types, the livers of 60 control and 60 ethanol-treated *Tg(hand2:EGFP;kdrl:ras-mCherry)* larvae were dissected immediately after ethanol treatment. The liver cell suspension was prepared as described (Yin et al., 2010). The *Tg(hand2:EGFP)*-positive cells, *Tg(kdrl:ras-mCherry)*-positive cells, and the remaining liver cells were sorted using a Becton Dickinson FACS ARIA 2 (BD Biosciences, San Jose, CA). Total RNA amplification and cDNA preparation were conducted on the HSC and endothelial cell samples by using a WT-Ovation Pico System (NuGEN Technologies; San Carlos, CA). cDNA from the parenchymal cell sample was synthesized by using SuperScript III First-Strand Synthesis System without amplification. 5 ng of HSC cDNA, 5 ng of endothelial cell cDNA and 100 ng of parenchymal cell cDNA were used in each qPCR reaction.

All qPCR experiments were conducted on QuantStudio6 or StepOnePlus qPCR Systems (Life Technologies). The relative expression of each gene was determined after normalization to the expression of the housekeeping gene *eef1a1l1* using the relative standard curve method (Larionov et al., 2005). Each qPCR reaction was run in triplicate. The post-data analyses were performed using GraphPad Prism software (GraphPad, La Jolla, CA). The optimized primers targeting each gene are listed in Table S1.

### Oil Red O stain and histologic analysis

Whole-mount Oil Red O stain was performed as described (Kim et al., 2015). For histologic analysis, larvae were fixed in 10% formalin and embedded in paraffin. 6 μm sections were stained with hematoxylin and eosin (H&E). Images were obtained on a Carl Zeiss Discovery V8 stereomicroscope using a Zeiss Axioncam MRc5 camera (Carl Zeiss, Jena, Germany).

### Overexpressing the active forms of Srebf1 and Srebf2

In human, the active form of SREBF1 contains amino acids 1-460 of the protein (Shimano et al., 1997) and the active form of SREBF2 contains amino acids 1-468 (J. Goldstein, UT Southwestern Medical Center, Dallas, Texas, USA, personal communication) (Shimano et al., 1997). We conducted protein alignment analysis and found that they correspond to amino acid 1-411 of Srebf1 and amino acid 1-445 of Srebf2 in zebrafish. The following PCR primers were used to amplify the corresponding coding sequences: *a-srebf1*, forward: 5′-ATGAATCTGTCTTTTGACGA-3′, reverse: 5′-TGAAGCTGGAGGAGTGGGGA-3′; *a-srebf2*, forward: 5′-ATGGACGCCTCGGAGTTTAT-3′, reverse: 5′-AGAGTCCGGCTCACTCTTCA-3′. Both coding sequences were cloned using the Gateway system into a vector that contains the *fabp10a* promoter to drive hepatocyte-specific expression and a 2A-ras-GFP tag for visualizing the cells that incorporate the transgene. A mixture of 70 pg *Tg(fabp10a:a-srebf1-2A-ras-GFP)* or *Tg(fabp10a:a-srebf2-2A-ras-GFP)* plasmid and 70 pg Tol2 transposase mRNA was injected into WT embryos at the 1-cell stage. Both uninjected control and injected larvae were treated with DMSO or 3 μM ZM between 123 and 147 hpf and fixed in 4% PFA immediately after the treatment. To detect lipids, the animals were stained in 500 ng/ml Nile Red (Life Technologies) dissolved in PBS for 2 h at room temperature before being imaged by confocal microscope.

### Statistical analysis

All statistical analyses were performed using GraphPad Prism. Statistical differences in the number of HSCs (Fig. 1J; Fig. 5F,G; Fig. 7C; Fig. S1F), the number of neutrophils and macrophages (Fig. S7E,J), the average diameter of the intrahepatic vessels (Fig. 3G), and gene expression levels (Fig. 2E-I; Fig. 4J; Fig. 5H,I; Fig. 6B-D; Fig. S3A,B; Fig. S4A-D; Fig. S5A) were analyzed by one-way ANOVA and Tukey's post-hoc test. Differences in the percentages of proliferating HSCs (Fig. 1L), the numbers of intrahepatic vessel branches (Fig. 3H,I; Fig. 5E; Fig. 7A), and the percentages of larvae with steatosis (Fig. 4I; Fig. 5J,K; Fig. 7H) were analyzed by two-tailed Student's *t*-test. Analysis of differences in the distribution of steatosis versus no steatosis (Fig. 4K,L) was performed by the contingency table Fisher's exact test. *P*-values less than 0.05 were considered statistically significant.
